# Supplementary material for: Effective Hydrogenation of 3-(2”-furyl)- and 3-(2”-thienyl)-1-(2’-hydroxyphenyl)-prop-2-en-1-one in Selected Yeast Cultures
Source: Molecules. 2019 Sep 2;24(17):3185. doi: 10.3390/molecules24173185 (PMC6749209; doi:10.3390/molecules24173185)

Supplementary Data

Effective Hydrogenation of 3-(2″-furyl)- and 3-(2″-thienyl)-1-(2′-hydroxyphenyl)-prop-2-en-1-one in Selected Yeast Cultures

Mateusz Łużny *, Martyna Krzywda, Ewa Kozłowska, Edyta Kostrzewa-Susłow and Tomasz Janeczko *

Department of Chemistry, Wrocław University of Environmental and Life Sciences, Norwida 25, 50-375 Wrocław, Poland

* Correspondence: mat.luzny@gmail.com(M.Ł.) janeczko13@interia.pl (T.J.); Tel.: +48-713-205-195 (T.J.)

Content

FigureS1. ^{1}H-NMR spectral of 3-(2”-furyl)-1-(2’-hydroxyphenyl)-prop-2-en-1-one (**1**) ($CDCl_3$, 600 MHz)

FigureS2. Part of the ^{1}H-NMR spectral of 3-(2”-furyl)-1-(2’-hydroxyphenyl)-prop-2-en-1-one (**1**) ($CDCl_3$, 600 MHz)

FigureS3. ^{13}C-NMR spectral of 3-(2”-furyl)-1-(2’-hydroxyphenyl)-prop-2-en-1-one (**1**) ($CDCl_3$, 151 MHz)

FigureS4. HSQC spectral of 3-(2”-furyl)-1-(2’-hydroxyphenyl)-prop-2-en-1-one (**1**) ($CDCl_3$, 151 MHz)

FigureS5. COSY spectral of 3-(2”-furyl)-1-(2’-hydroxyphenyl)-prop-2-en-1-one (**1**) ($CDCl_3$, 600 MHz)

FigureS6. ^{1}H-NMR spectral of 3-(2”-furyl)-1-(2’-hydroxyphenyl)-propan-1-one (**2**) ($CDCl_3$, 600 MHz)

FigureS7. Part of the ^{1}H-NMR spectral of 3-(2”-furyl)-1-(2’-hydroxyphenyl)- propan-1-one (**2**) ($CDCl_3$, 600 MHz)

FigureS8. ^{13}C-NMR spectral of 3-(2”-furyl)-1-(2’-hydroxyphenyl)- propan-1-one (**2**) ($CDCl_3$, 151 MHz)

FigureS9. HSQC spectral of 3-(2”-furyl)-1-(2’-hydroxyphenyl)- propan-1-one (**2**) ($CDCl_3$, 151 MHz)

FigureS10. COSY spectral of 3-(2”-furyl)-1-(2’-hydroxyphenyl)- propan-1-one (**2**) ($CDCl_3$, 600 MHz)

FigureS11. ^{1}H-NMR spectral of 3-(2”-thienyl)-1-(2’-hydroxyphenyl)-prop-2-en-1-one (**3**) ($CDCl_3$, 600 MHz)

FigureS12. Part of the ^{1}H-NMR spectral of 3-(2”-thienyl)-1-(2’-hydroxyphenyl)-prop-2-en-1-one (**3**) ($CDCl_3$, 600 MHz)

FigureS13. ^{13}C-NMR spectral of 3-(2”-thienyl)-1-(2’-hydroxyphenyl)-prop-2-en-1-one (**3**) ($CDCl_3$, 151 MHz)

FigureS14. HSQC spectral of 3-(2”-thienyl)-1-(2’-hydroxyphenyl)-prop-2-en-1-one (**3**) ($CDCl_3$, 151 MHz)

FigureS15. COSY spectral of 3-(2”-thienyl)-1-(2’-hydroxyphenyl)-prop-2-en-1-one (**3**) ($CDCl_3$, 600 MHz)

FigureS16. HMBC spectral of 3-(2”-thienyl)-1-(2’-hydroxyphenyl)-prop-2-en-1-one (**3**) ($CDCl_3$, 151 MHz)

FigureS17. ^{1}H-NMR spectral of 3-(2”-thienyl)-1-(2’-hydroxyphenyl)-propan-1-one (**4**) ($CDCl_3$, 600 MHz)

FigureS18. Part of the ^{1}H-NMR spectral of 3-(2”-thienyl)-1-(2’-hydroxyphenyl)- propan-1-one (**4**) ($CDCl_3$, 600 MHz)

FigureS19. ^{13}C-NMR spectral of 3-(2”-thienyl)-1-(2’-hydroxyphenyl)- propan-1-one (**4**) ($CDCl_3$, 151 MHz)

FigureS20. HSQC spectral of 3-(2”-thienyl)-1-(2’-hydroxyphenyl)- propan-1-one (**4**) ($CDCl_3$, 151 MHz)

FigureS21. COSY spectral of 3-(2”-thienyl)-1-(2’-hydroxyphenyl)- propan-1-one (**4**) ($CDCl_3$, 600 MHz)

FigureS22. HMBC spectral of 3-(2”-thienyl)-1-(2’-hydroxyphenyl)- propan-1-one (**4**) ($CDCl_3$, 151 MHz)

FigureS23. Chromatogram presenting the composition of reaction mixture after 1 hour incubation of **1** in the culture of the *Saccharomyces cerevisiae* KCh 464 strain.

FigureS24. Chromatogram presenting the composition of reaction mixture after 3 hours incubation of **1** in the culture of the *Saccharomyces cerevisiae* KCh 464 strain.

FigureS25. Chromatogram presenting the composition of reaction mixture after 6 hours incubation of **1** in the culture of the *Saccharomyces cerevisiae* KCh 464 strain.

FigureS26. Chromatogram presenting the composition of reaction mixture after 12 hours incubation of **1** in the culture of the *Saccharomyces cerevisiae* KCh 464 strain.

FigureS27. Chromatogram presenting the composition of reaction mixture after 1 hour incubation of **3** in the culture of the *Saccharomyces cerevisiae* KCh 464 strain.

FigureS28. Chromatogram presenting the composition of reaction mixture after 3 hours incubation of **3** in the culture of the *Saccharomyces cerevisiae* KCh 464 strain.

FigureS29. Chromatogram presenting the composition of reaction mixture after 6 hours incubation of **3** in the culture of the *Saccharomyces cerevisiae* KCh 464 strain.

FigureS30. Chromatogram presenting the composition of reaction mixture after 12 hours incubation of **3** in the culture of the *Saccharomyces cerevisiae* KCh 464 strain.

FigureS1. ^{1}H-NMR spectral of 3-(2”-furyl)-1-(2’-hydroxyphenyl)-prop-2-en-1-one (**1**) ($CDCl_3$, 600 MHz)

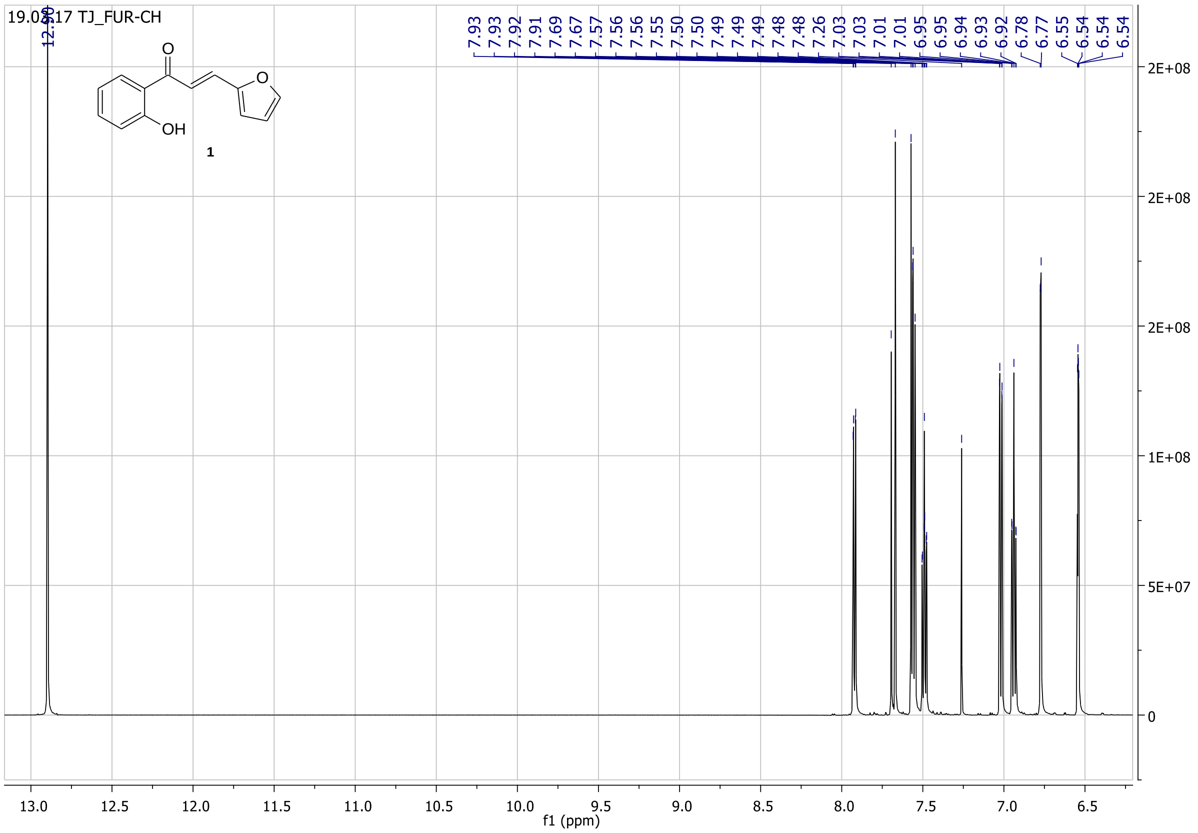

FigureS2. Part of the ^{1}H-NMR spectral of 3-(2”-furyl)-1-(2’-hydroxyphenyl)-prop-2-en-1-one (**1**) ($CDCl_3$, 600 MHz)

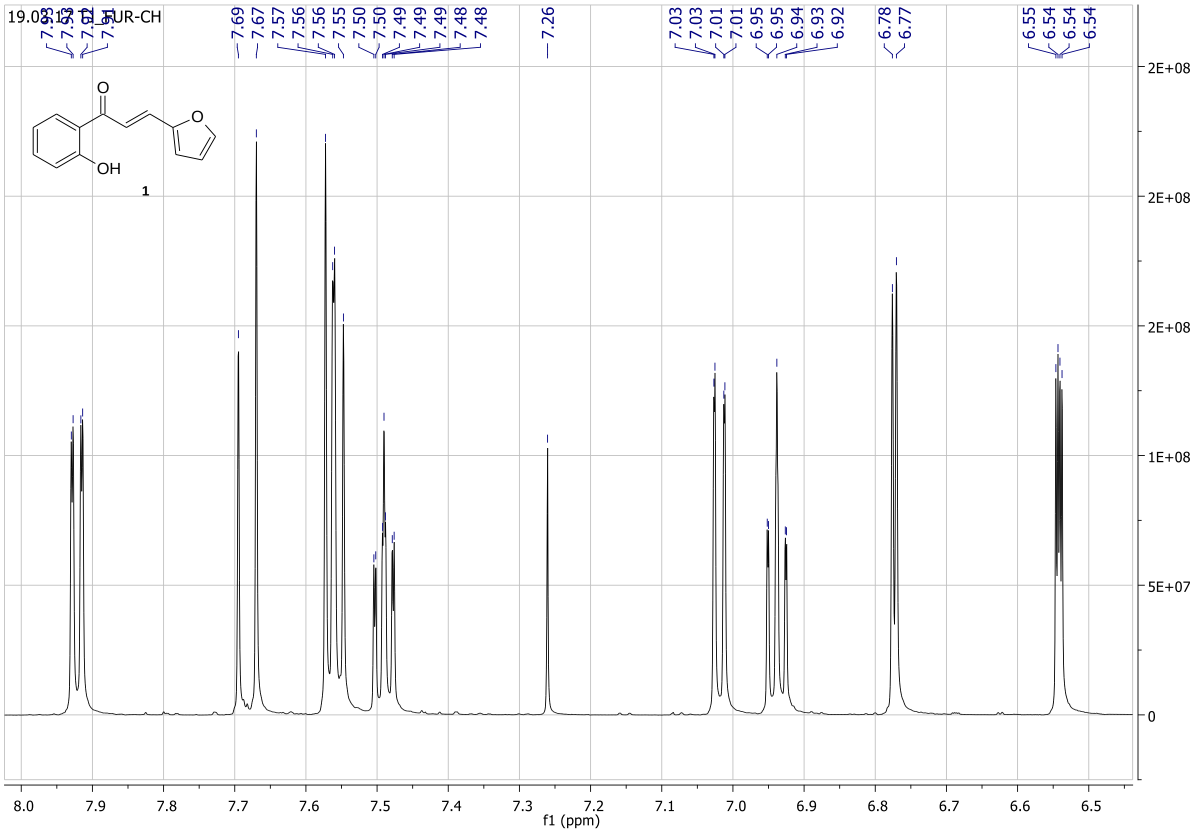

FigureS3. ^{13}C-NMR spectral of 3-(2”-furyl)-1-(2’-hydroxyphenyl)-prop-2-en-1-one (**1**) ($CDCl_3$, 151 MHz)

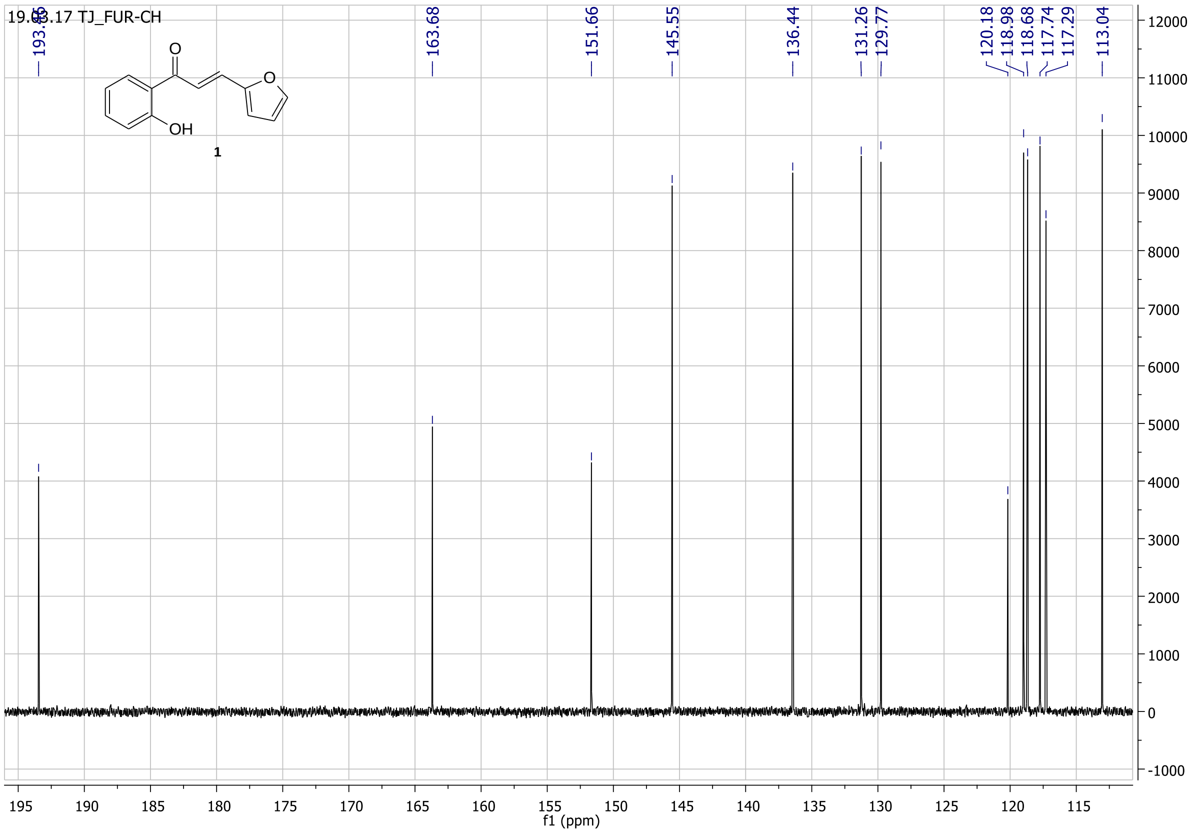

FigureS4. HSQC spectral of 3-(2”-furyl)-1-(2’-hydroxyphenyl)-prop-2-en-1-one (**1**) ($CDCl_3$, 151 MHz)

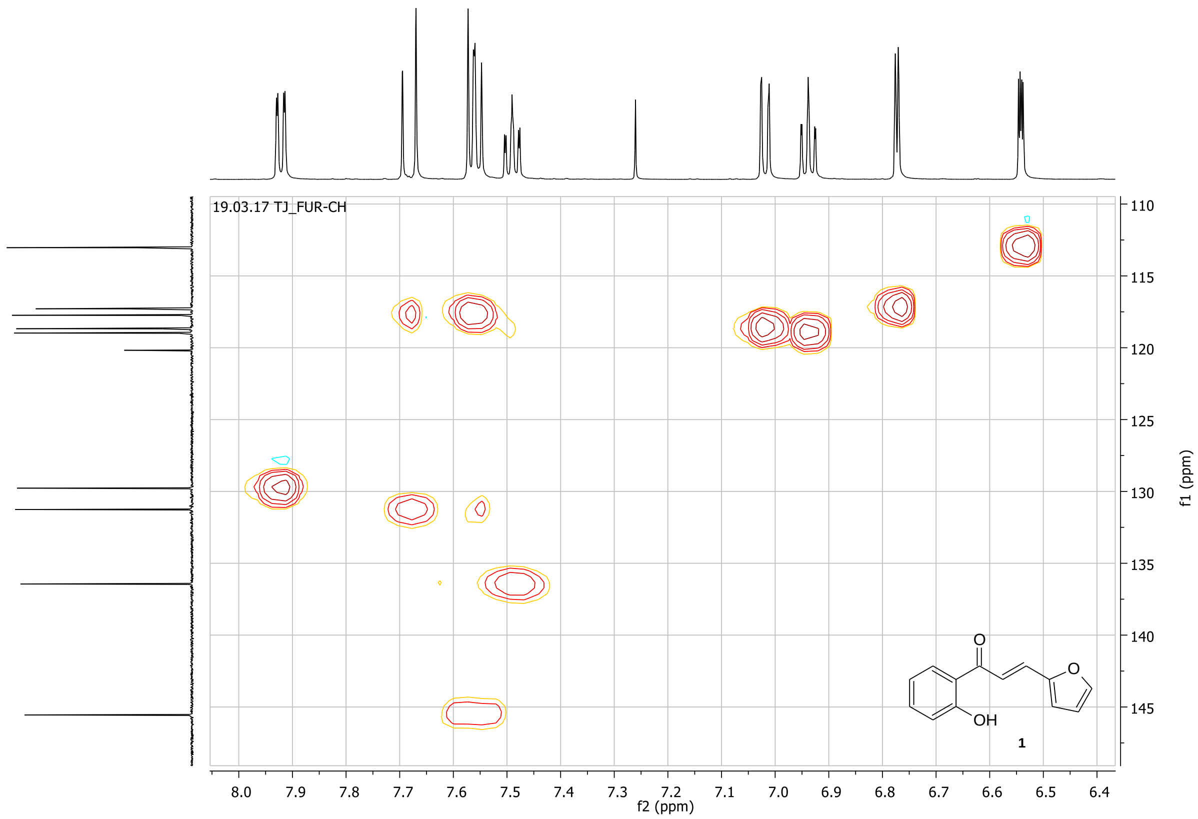

FigureS5. COSY spectral of 3-(2''-furyl)-1-(2'-hydroxyphenyl)-prop-2-en-1-one (**1**) ($CDCl_3$, 600 MHz)

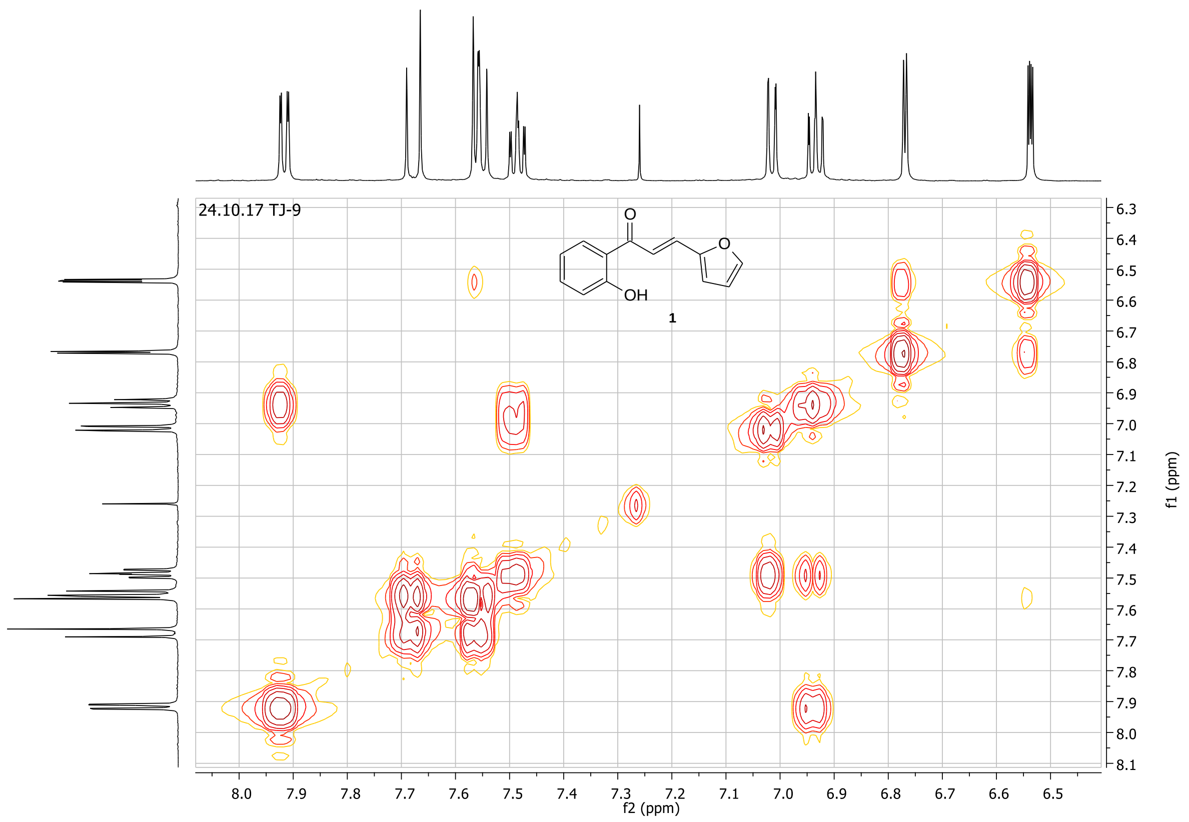

FigureS6. ^{1}H-NMR spectral of 3-(2''-furyl)-1-(2'-hydroxyphenyl)-propan-1-one (**2**) ($CDCl_3$, 600 MHz)

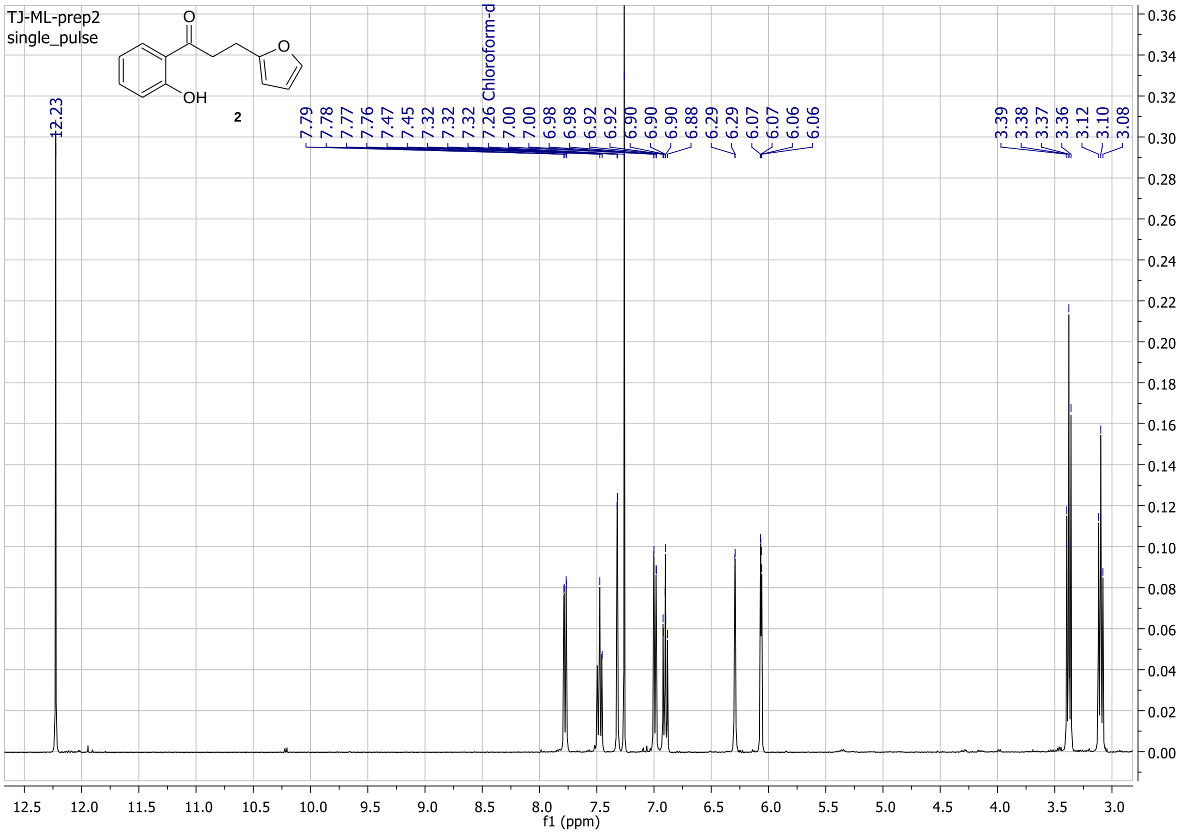

FigureS7. Part of the ^{1}H-NMR spectral of 3-(2”-furyl)-1-(2’-hydroxyphenyl)- propan-1-one (**2**) ($CDCl_3$, 600 MHz)

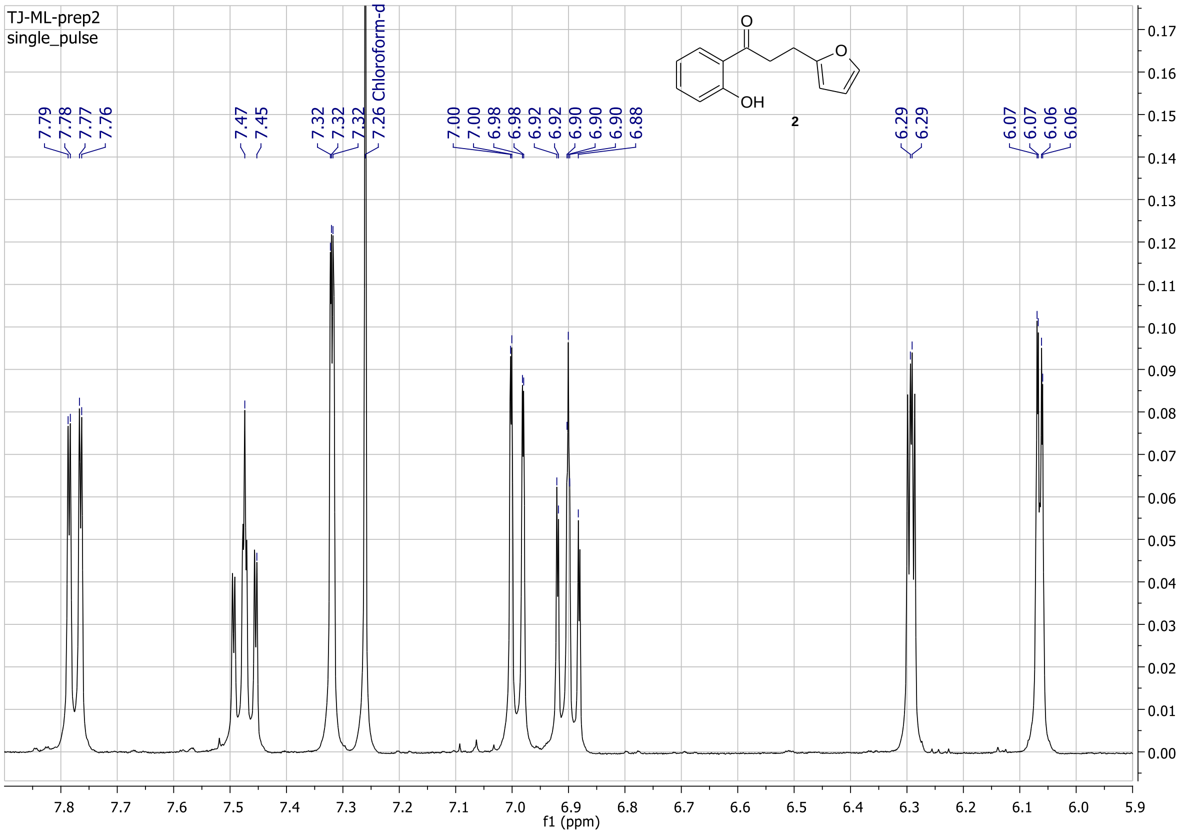

FigureS8. ^{13}C-NMR spectral of 3-(2”-furyl)-1-(2’-hydroxyphenyl)- propan-1-one (**2**) ($CDCl_3$, 151 MHz)

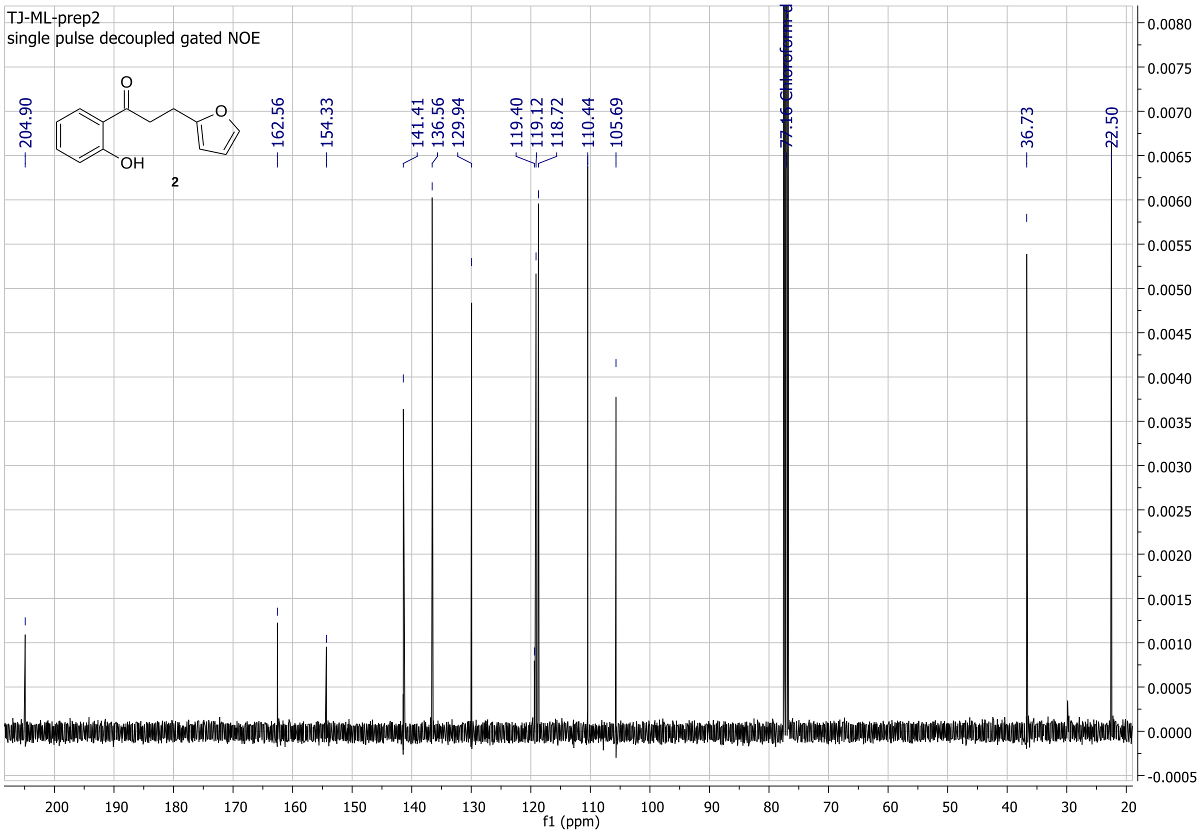

FigureS9. HSQC spectral of 3-(2”-furyl)-1-(2’-hydroxyphenyl)- propan-1-one (**2**) ($CDCl_3$, 151 MHz)

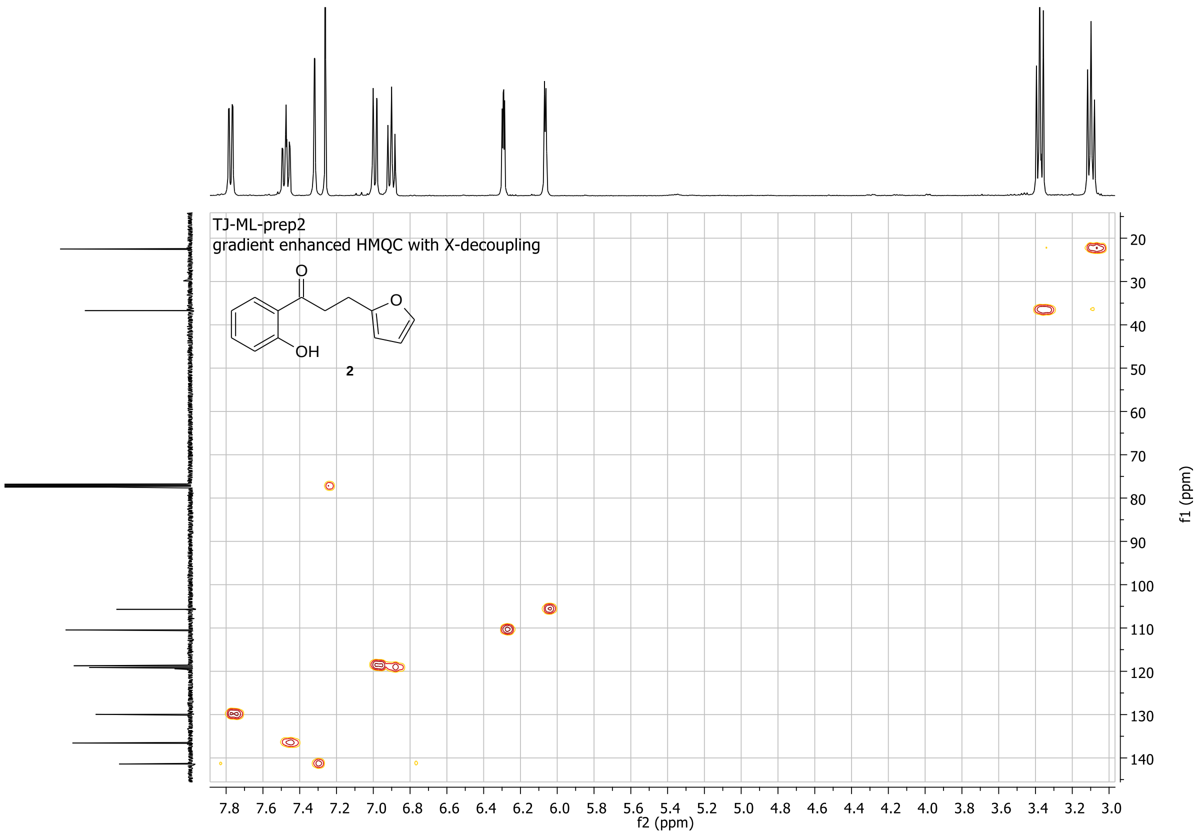

FigureS10. COSY spectral of 3-(2”-furyl)-1-(2’-hydroxyphenyl)- propan-1-one (**2**) ($CDCl_3$, 600 MHz)

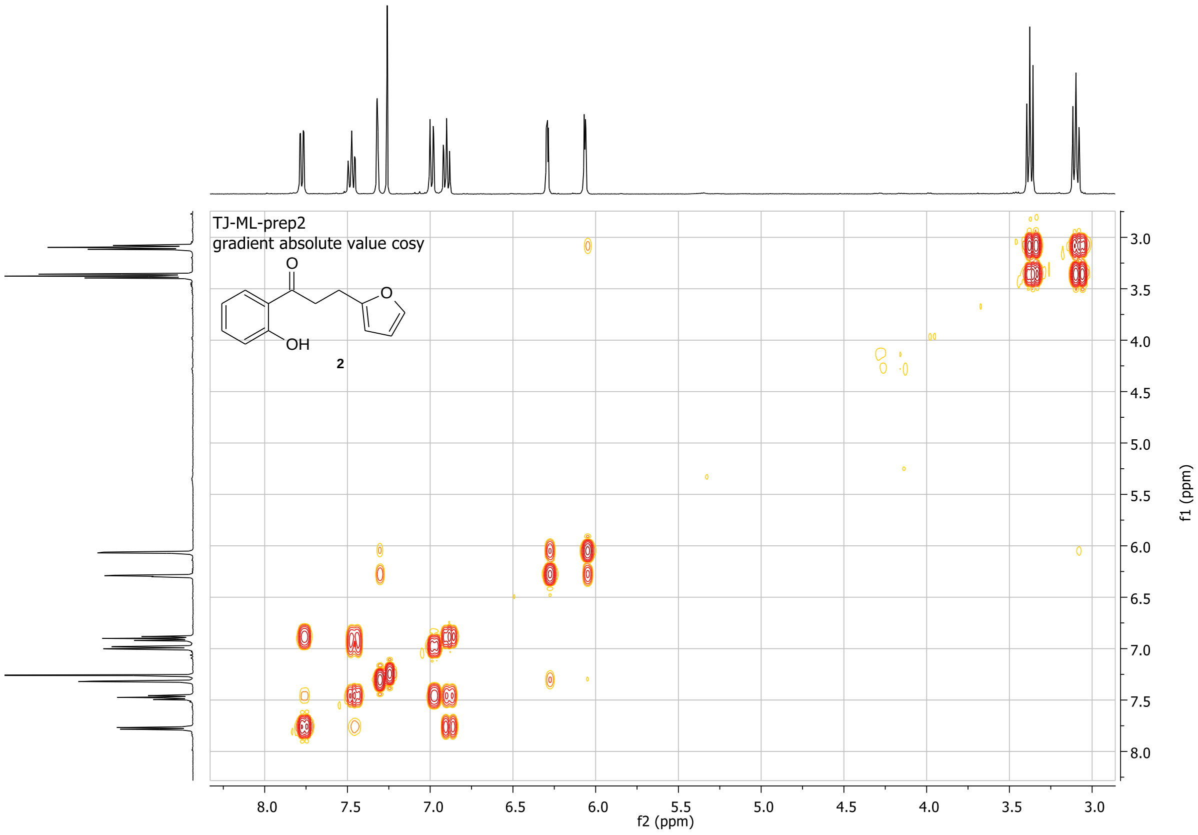

FigureS11. ^{1}H-NMR spectral of 3-(2”-thienyl)-1-(2’-hydroxyphenyl)-prop-2-en-1-one (**3**) ($CDCl_3$, 600 MHz)

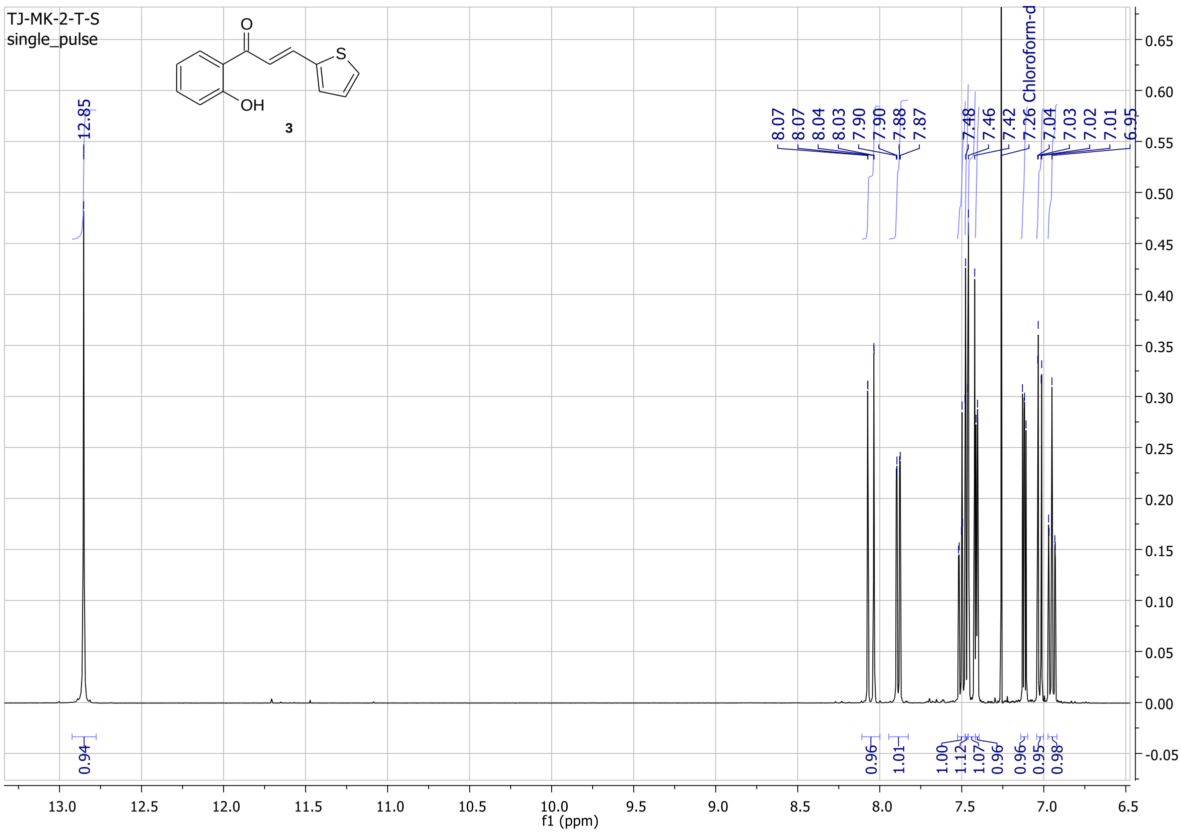

FigureS12. Part of the ^{1}H-NMR spectral of 3-(2”-thienyl)-1-(2’-hydroxyphenyl)-prop-2-en-1-one (**3**) ($CDCl_3$, 600 MHz)

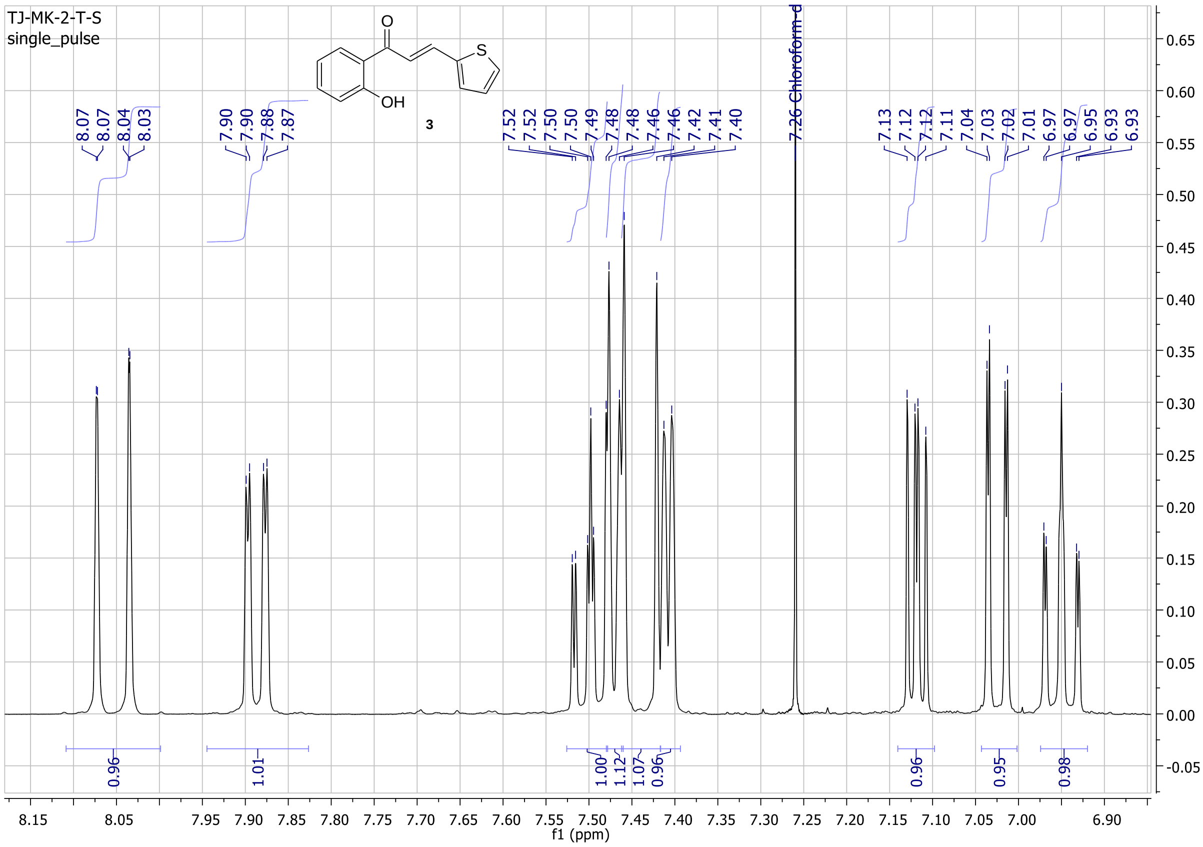

FigureS13. ^{13}C-NMR spectral of 3-(2''-thienyl)-1-(2'-hydroxyphenyl)-prop-2-en-1-one (**3**) ($CDCl_3$, 151 MHz)

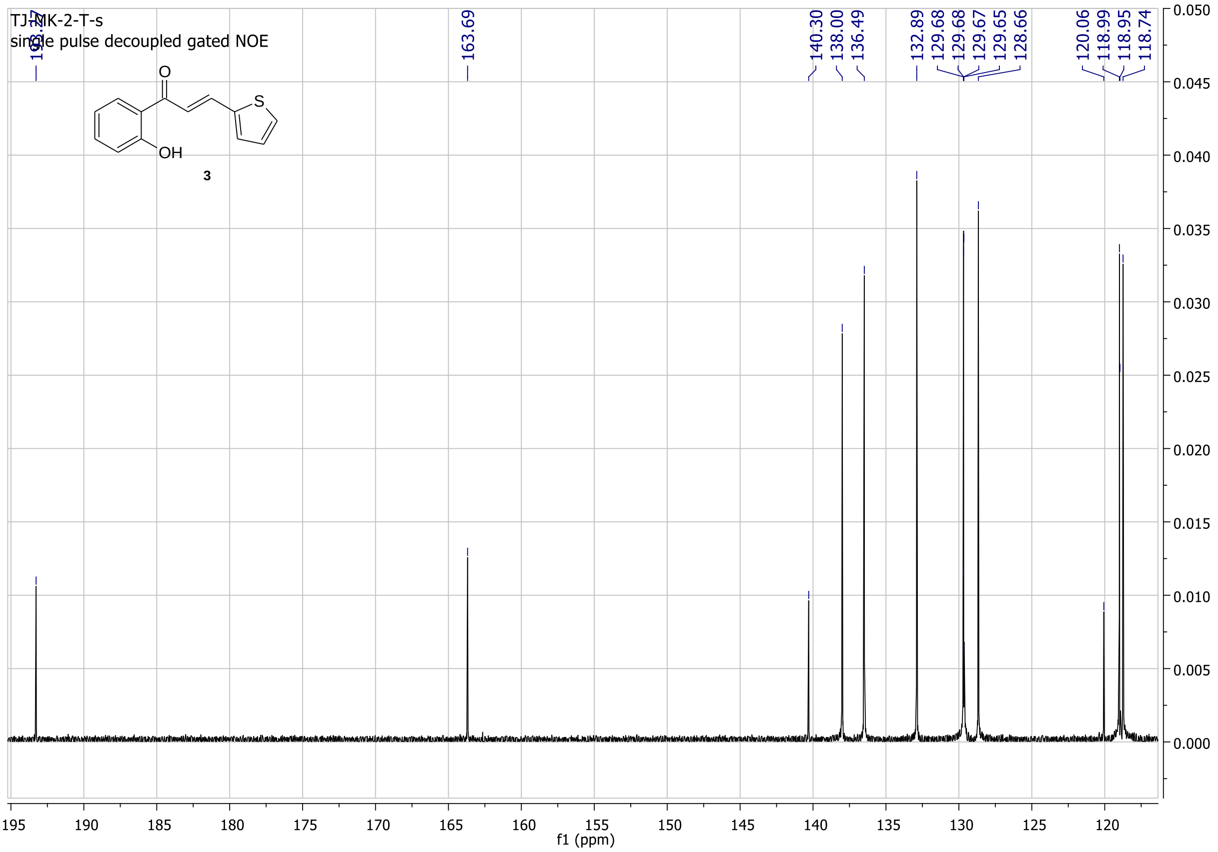

FigureS14. HSQC spectral of 3-(2''-thienyl)-1-(2'-hydroxyphenyl)-prop-2-en-1-one (**3**) ($CDCl_3$, 151 MHz)

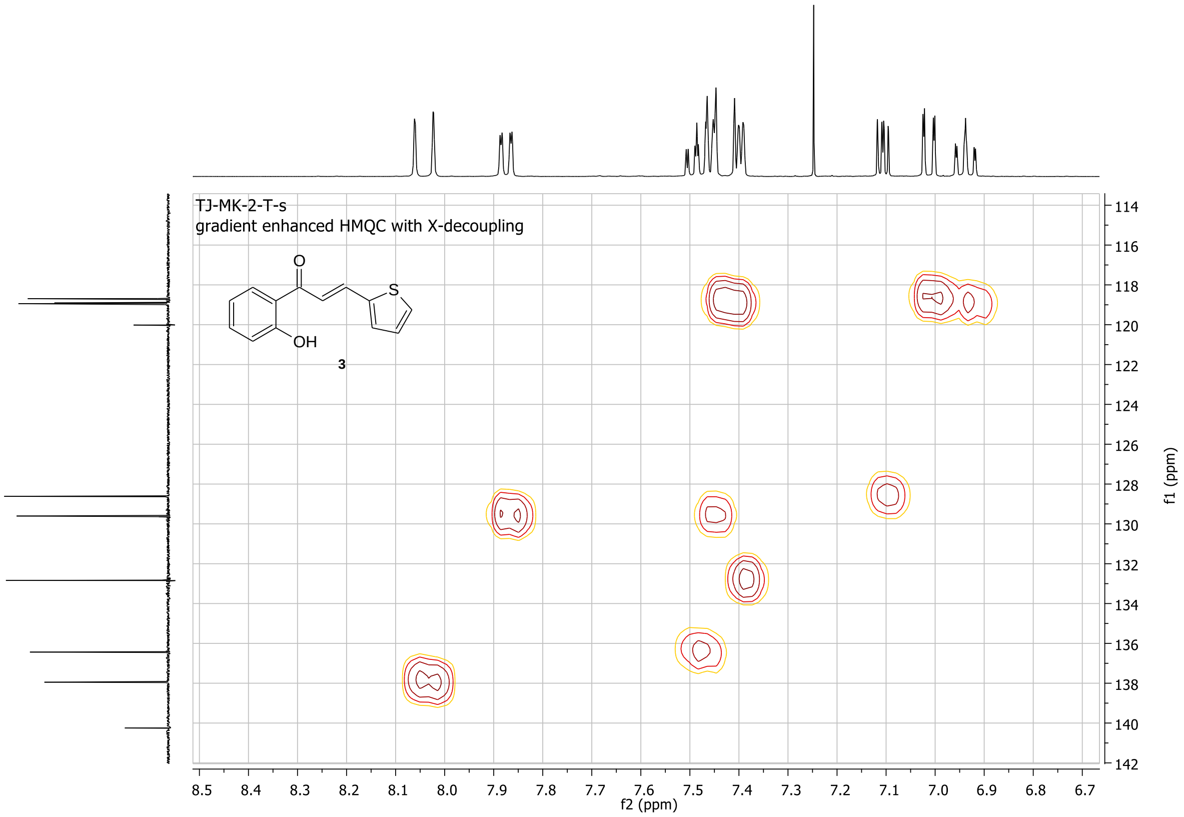

FigureS15. COSY spectral of 3-(2”-thienyl)-1-(2’-hydroxyphenyl)-prop-2-en-1-one (**3**) ($CDCl_3$, 600 MHz)

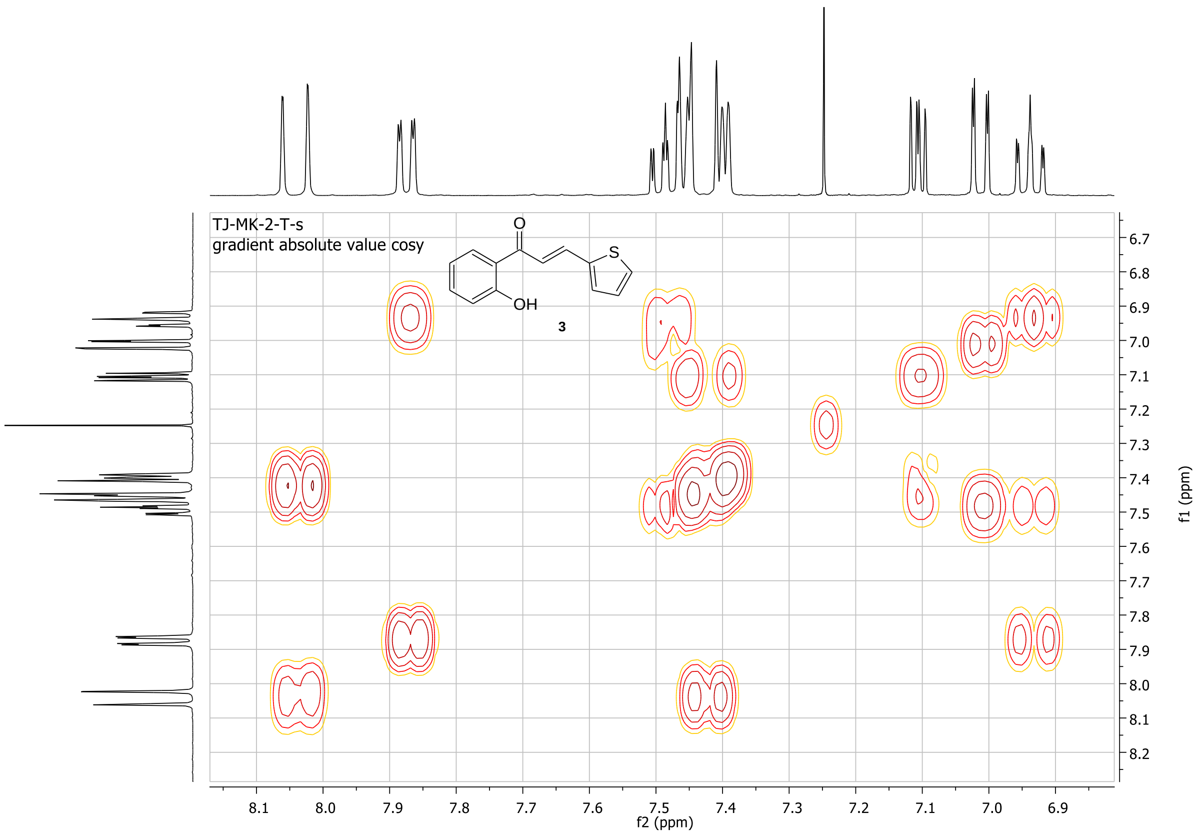

FigureS16. HMBC spectral of 3-(2”-thienyl)-1-(2’-hydroxyphenyl)-prop-2-en-1-one (**3**) ($CDCl_3$, 151 MHz)

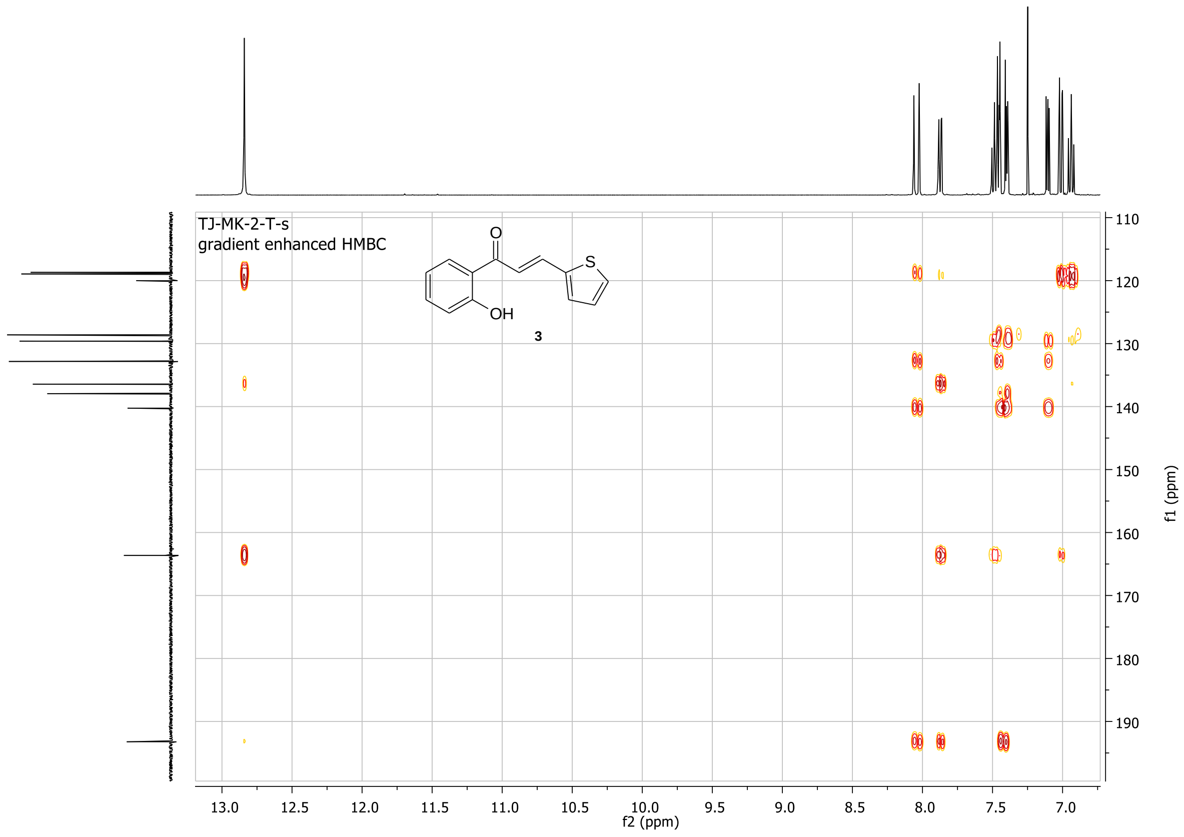

FigureS17. ^{1}H-NMR spectral of 3-(2”-thienyl)-1-(2’-hydroxyphenyl)-propan-1-one (**4**) ($CDCl_3$, 600 MHz)

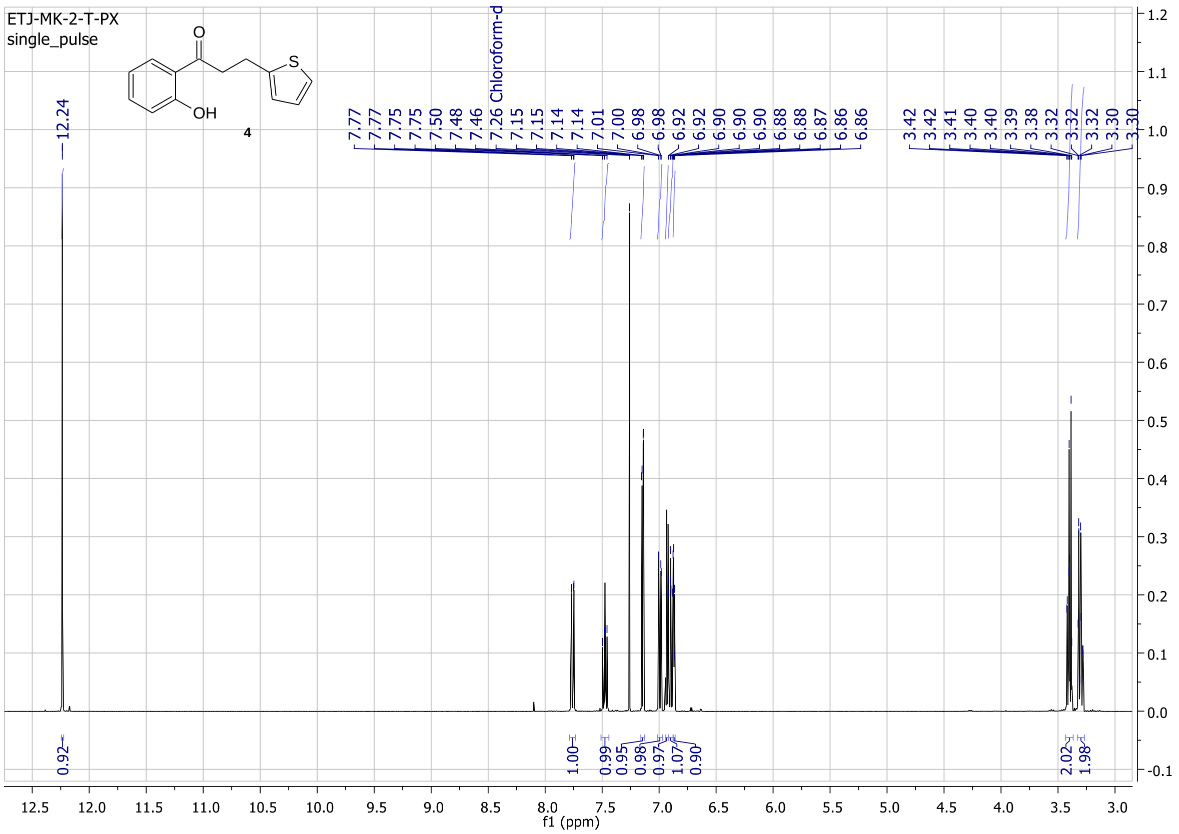

FigureS18. Part of the ^{1}H-NMR spectral of 3-(2”-thienyl)-1-(2’-hydroxyphenyl)- propan-1-one (**4**) ($CDCl_3$, 600 MHz)

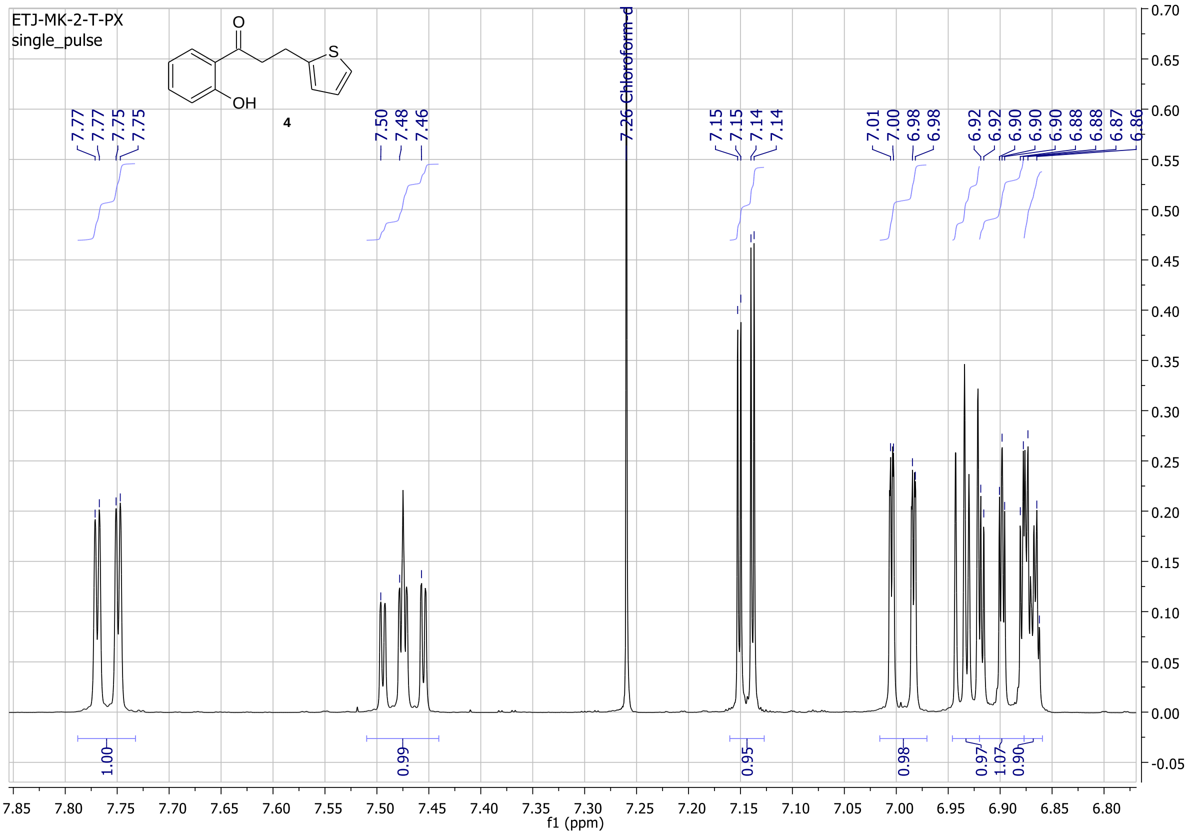

FigureS19. ^{13}C-NMR spectral of 3-(2”-thienyl)-1-(2’-hydroxyphenyl)- propan-1-one (**4**) ($CDCl_3$, 151 MHz)

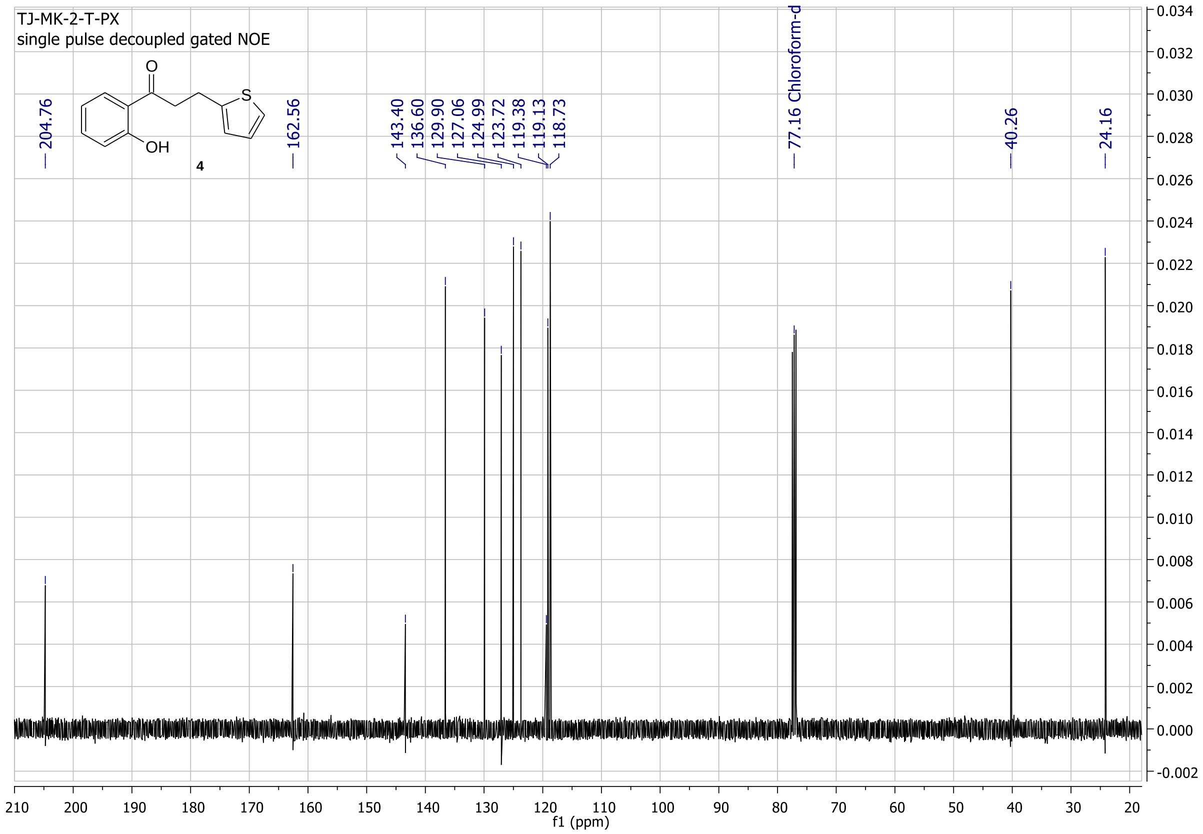

FigureS20. HSQC spectral of 3-(2”-thienyl)-1-(2’-hydroxyphenyl)- propan-1-one (**4**) ($CDCl_3$, 151 MHz)

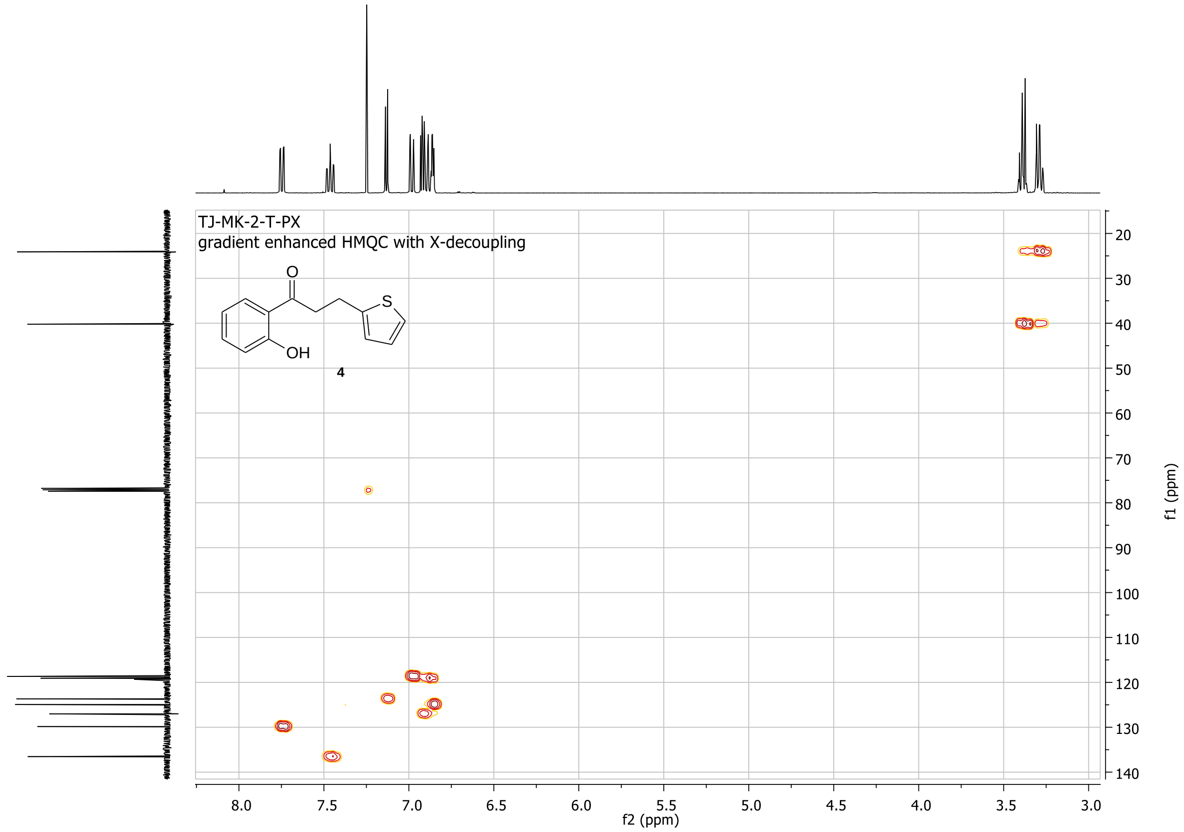

FigureS21. COSY spectral of 3-(2”-thienyl)-1-(2’-hydroxyphenyl)- propan-1-one (**4**) ($CDCl_3$, 600 MHz)

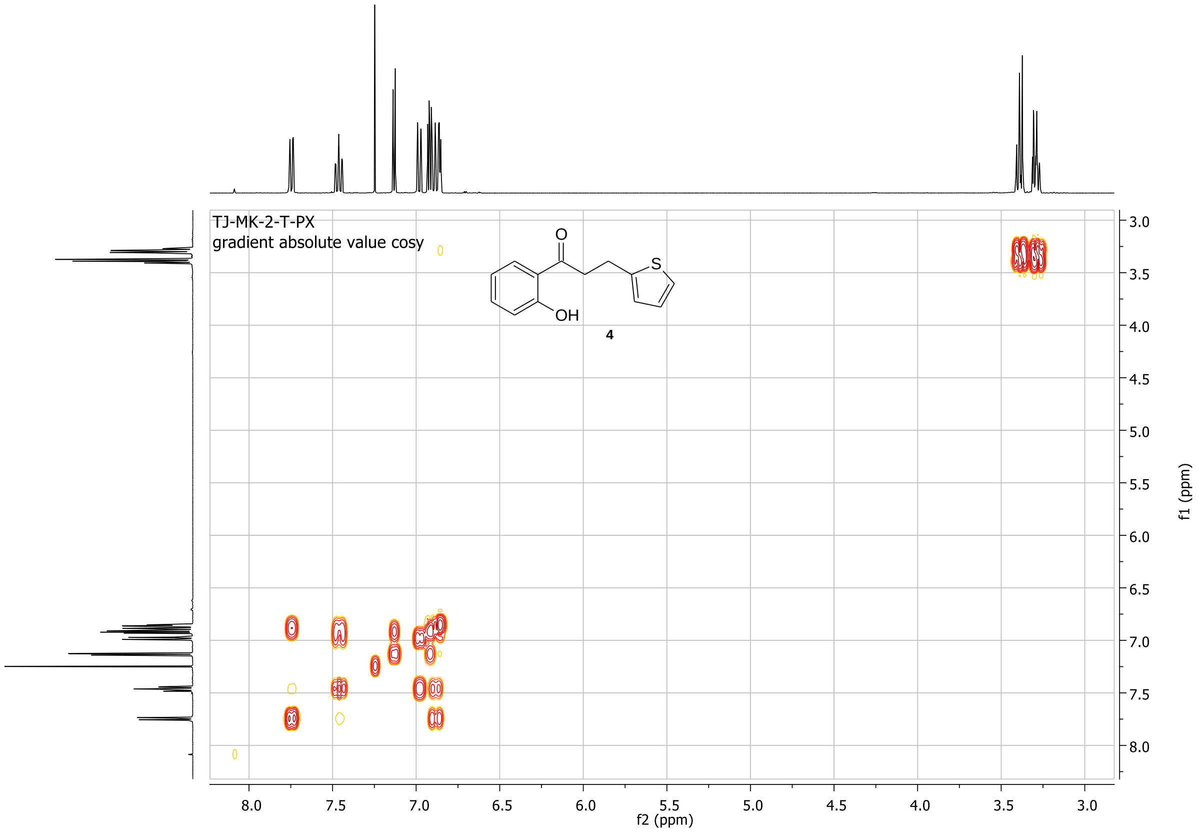

FigureS22. HMBC spectral of 3-(2”-thienyl)-1-(2’-hydroxyphenyl)- propan-1-one (**4**) ($CDCl_3$, 151 MHz)

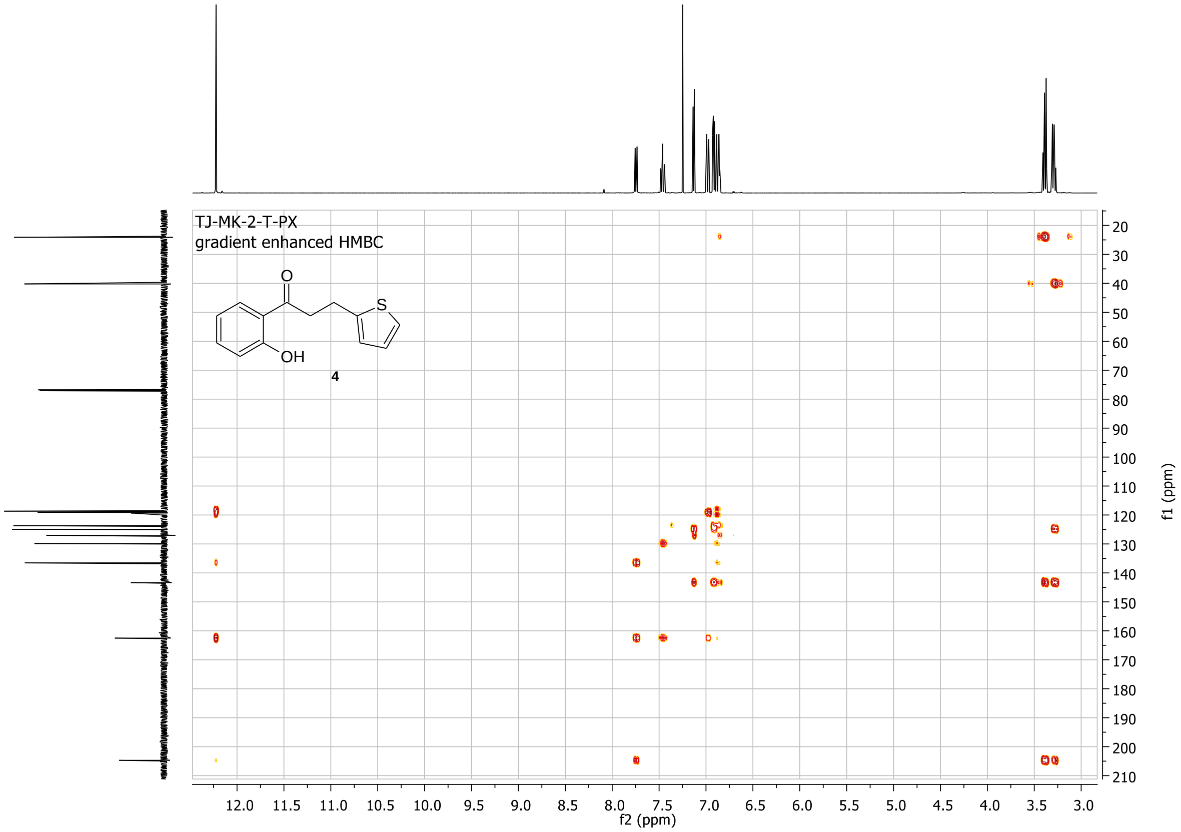

Selected chromatograms of biotransformation course:

FigureS23. Chromatogram presenting the composition of reaction mixture after 1 hour incubation of **1** in the culture of the *Saccharomyces cerevisiae* KCh 464 strain.

Retention time of the substrate (3-(2”-furyl)-1-(2’-hydroxyphenyl)-prop-2-en-1-one (**1**)) – 9.6min and product (3-(2”-furyl)-1-(2’-hydroxyphenyl)- propan-1-one (**2**)) - 8.5min.

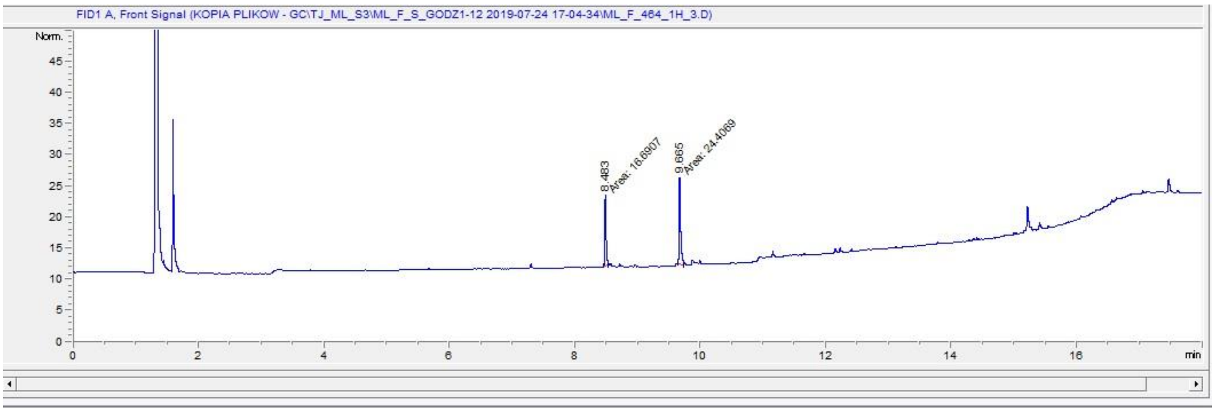

FigureS24. Chromatogram presenting the composition of reaction mixture after 3 hours incubation of **1** in the culture of the *Saccharomyces cerevisiae* KCh 464 strain.

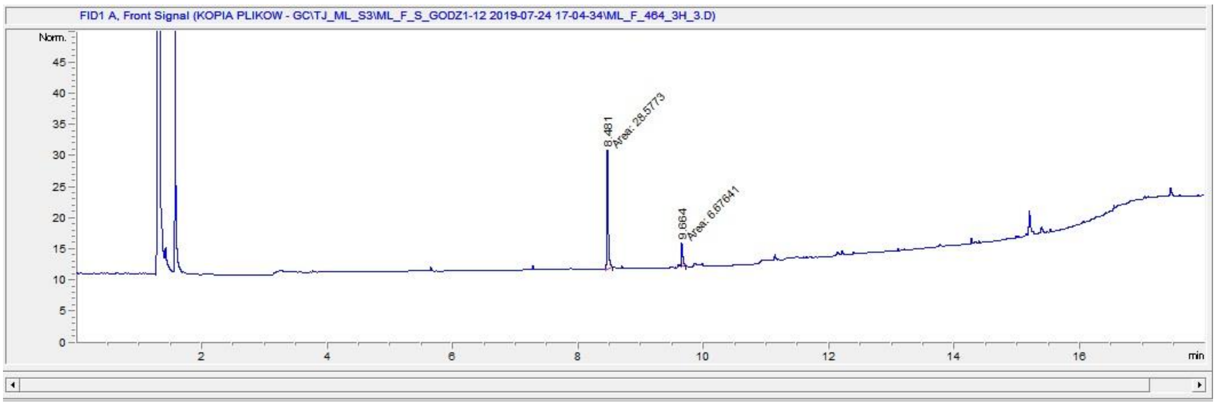

FigureS25. Chromatogram presenting the composition of reaction mixture after 6 hours incubation of **1** in the culture of the *Saccharomyces cerevisiae* KCh 464 strain.

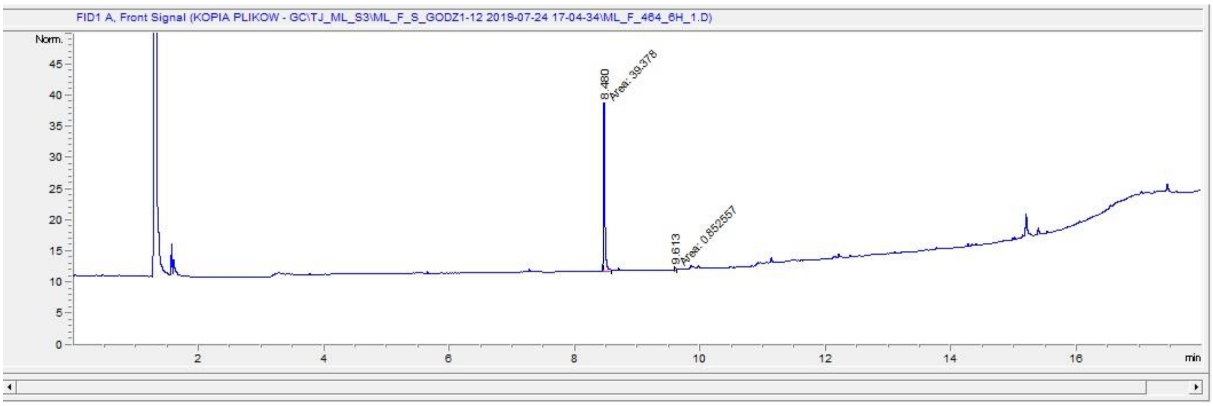

FigureS26. Chromatogram presenting the composition of reaction mixture after 12 hours incubation of **1** in the culture of the *Saccharomyces cerevisiae* KCh 464 strain.

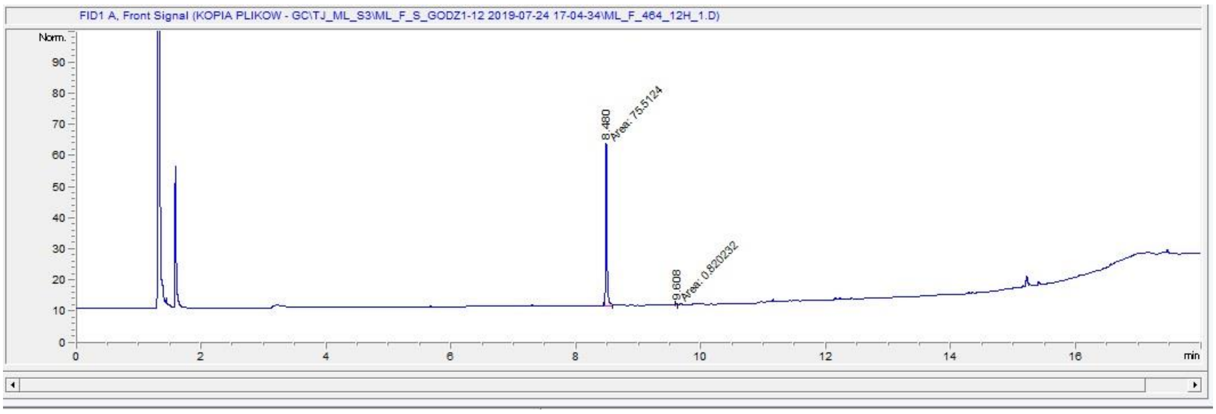

FigureS27. Chromatogram presenting the composition of reaction mixture after 1 hour incubation of **3** in the culture of the *Saccharomyces cerevisiae* KCh 464 strain.

Retention time of the substrate (3-(2”-thienyl)-1-(2’-hydroxyphenyl)-prop-2-en-1-one (**3**)) – 10.8min and product (3-(2”-thienyl)-1-(2’-hydroxyphenyl)- propan-1-one (4)) - 9.7min.

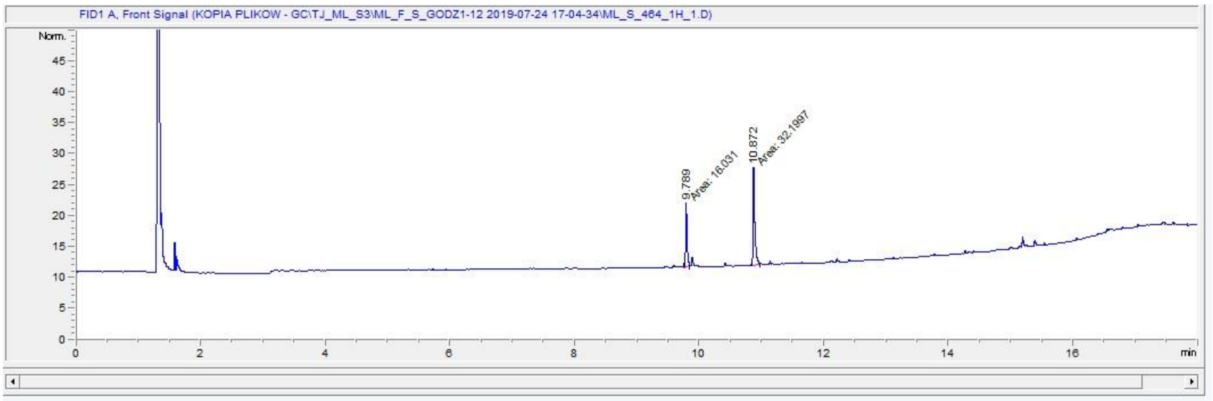

FigureS28. Chromatogram presenting the composition of reaction mixture after 3 hours incubation of **3** in the culture of the *Saccharomyces cerevisiae* KCh 464 strain.

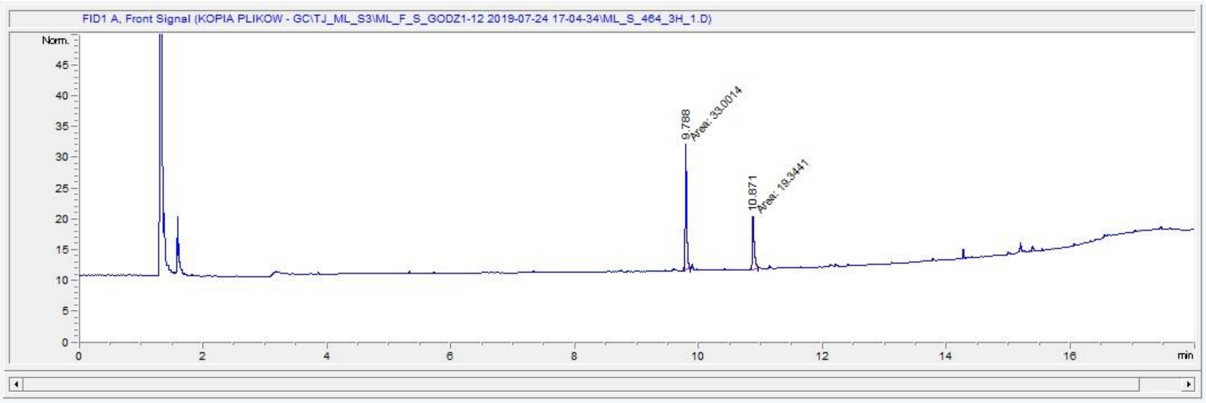

FigureS29. Chromatogram presenting the composition of reaction mixture after 6 hours incubation of **3** in the culture of the *Saccharomyces cerevisiae* KCh 464 strain.

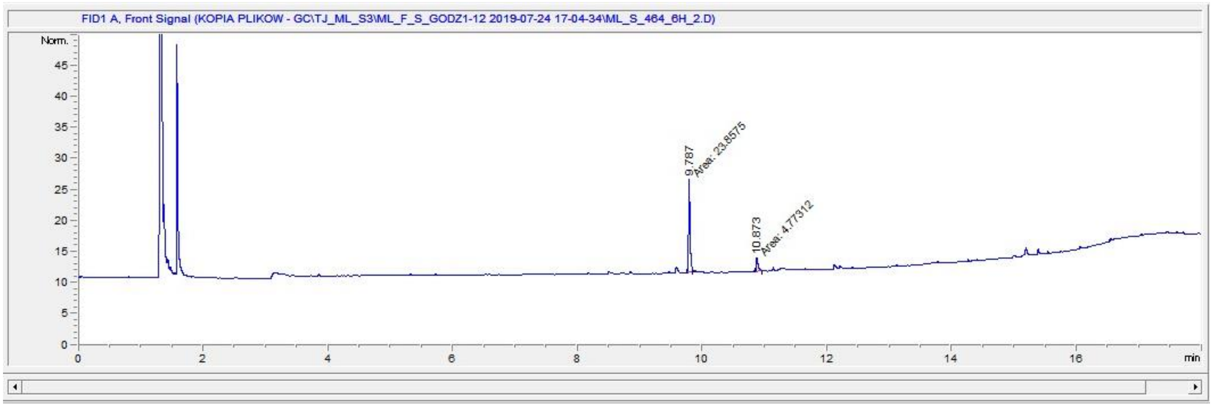

FigureS30. Chromatogram presenting the composition of reaction mixture after 12 hours incubation of **3** in the culture of the *Saccharomyces cerevisiae* KCh 464 strain.

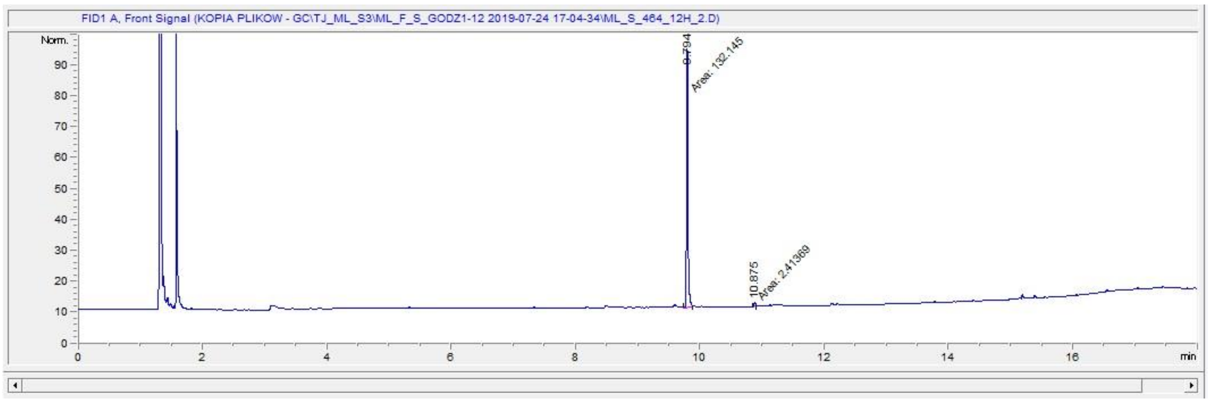

Supplement: Supplementary file 1 [file molecules-24-03185-s001.pdf]
